# Re-conceptualizing implementation outcomes of health innovations as modes or attributes: an integrated framework

**DOI:** 10.3389/frhs.2025.1373429

**Published:** 2025-05-22

**Authors:** Zuhur Balayah, Charitini Stavropoulou, Harry Scarbrough, Amit Nigam, Alexandra Ziemann

**Affiliations:** ^1^ Centre for Healthcare Innovation Research (CHIR), City St George's, University of London, London, United Kingdom; ^2^ Faculty of Management, Bayes Business School, City St George's, University of London, London, United Kingdom; ^3^ Department of Health Services Research and Management, School of Health and Psychological Sciences, City St George's, University of London, London, United Kingdom; ^4^ Department of Social and Policy Sciences, University of Bath, Bath, United Kingdom

**Keywords:** implementation frameworks, outcomes, conceptualization, innovations, integration, de-implementation, depth, breadth

## Abstract

The implementation of innovations in practice is challenging and often produces disappointing outcomes. Although the reasons for this are multifaceted, part of the challenge derives from the lack of consensus on how such implementation outcomes should be conceptualized and measured. In this review, we used a meta-ethnographic approach to enhance our theoretical conceptualization of implementation outcomes. By situating such outcomes within the overall process of implementation, we were able to unpack them analytically as the product of two major components, which we term “modes” and “attributes,” respectively. Modes comprise engagement, active implementation, and integration to foreground focal implementation outcomes. The attributes associated with the modes comprise implementation depth, implementation breadth, implementation pace, implementation adaptation, and de-implementation to indicate the features of the modes of implementation outcomes. Taken together, our analysis based on modes and attributes provides an integrated framework of implementation outcomes. The proposed framework enhances our understanding of the way in which implementation outcomes have been conceptualized in previous literature, enabling us to clarify the relations and distinctions between them in terms of translatability and complementarity. The proposed framework thus extends the conceptualization of implementation outcomes to better align with the complex reality of implementation practice, offering useful insights to researchers, practitioners, and policymakers.

## 1 Introduction

It has been argued that in healthcare, “without good implementation, innovation amounts to very little” (1). Yet, several healthcare innovations often fail to realize their potential because they are not implemented effectively or sustainably over time. This challenge has been observed in health systems globally (2–4), leading to significant waste of resources and energies.

Part of the explanation of why innovations fail to be implemented in practice is the lack of consensus on how individual implementation outcomes are conceptualized and measured (5, 6), making it challenging to learn about the reasons for both implementation successes and failures. Collectively, implementation outcomes have been defined, for example, as “success or failure of implementation” (7), “implementation effectiveness” (8), “impact of the implementation strategy” (9), or “effects of deliberate and purposive actions to implement new interventions” (10). In this review, we broadly refer to implementation outcomes as the consequences or effects of implementation efforts for innovations. Implementation outcomes (the focus of our review) are distinguished from other possible outcomes that result from implementing a given innovation, commonly referred to as service outcomes and client/clinical outcomes (10).

Over the last two decades, significant efforts have been made to develop and conceptualize frameworks that provide structure for evaluating implementation (11). Two seminal works that have become gold standards for assessing implementation effectiveness—the Implementation Outcomes Framework (IOF) (10) and Reach, Effectiveness/Efficacy, Adoption, Implementation, and Maintenance (RE-AIM) framework (12)—have been critical to the conceptualization of implementation outcomes. Researchers and practitioners have widely used both frameworks to evaluate implementation effectiveness (9, 13–15). For instance, other researchers have further developed implementation outcome instruments based on the IOF to establish measures matching these outcomes (16–18). The IOF is also included in the reporting standards and guidelines for implementation studies (9).

Yet, existing implementation outcome frameworks are not without limitations. First, prior research has emphasized the need for a broader conceptualization that includes, for example, dissemination outcomes, service implementation efficiency (8, 19), adaptation (13), and readiness as a precursor to early implementation outcomes (20). Expanding the conceptualization of implementation outcomes might also mean considering aspects, such as outcomes that refer to longer-term sustainability, spread and diffusion to other settings (21), and de-implementation (22). Second, reporting of implementation outcomes rarely considers their measurement attributes (5). Finally, there has also been some criticism regarding the practicability of the existing outcome measures for practice and policy stakeholders (5). As Martinez et al. (6) pointed out, whether implementation frameworks define different outcomes in the same way or define the same outcomes in different ways, they risk compromising a construct's validity and undermining the cross-research comparability of results.

This review aims to map and consolidate existing conceptualizations of implementation outcomes to develop an integrated framework. We use an interpretive synthesis approach to systematically compare how implementation outcomes have been conceptualized across multiple disciplinary perspectives to harmonize the conceptual meanings of these outcomes. We do this by considering the relatedness of outcome terminologies and conceptual definitions to elucidate their translatability, distinctions, and partial overlaps and identify how these elements contribute to explain components of a broader conceptual meaning and its associated characteristics.

Our paper contributes to both the academic literature and policy debate. More than a decade since the publication of IOF (10) and over two decades since the publication of RE-AIM (12), an integrated and disambiguated (re)conceptualization of implementation outcomes can help counter this divergence in the operationalization of outcomes, enable a more holistic and comprehensive understanding of implementation effectiveness or failure, and clarify how de-implementation and innovation diffusion/spread efforts fit with normative implementation evaluations. Harmonizing implementation outcomes terminology is crucial to support practitioners and policymakers in implementing innovation more effectively (15).

## 2 Methodology: interpretive synthesis review

Unlike conventional systematic review questions, our review question was loosely formulated, first, to consolidate conceptualizations of implementation outcomes and, second, to go beyond mere synthesis and provide holistic interpretations and re-conceptualization of implementation outcomes. To do this, we used a meta-ethnography approach (23) as the main approach, combined with the Behaviour or phenomenon of interest, Health context, Exclusions, Models, and Theories (BeHEMoTh) approach (24) for searching and selecting the relevant literature in the first instance.

Meta-ethnography is one of several approaches to undertaking interpretive synthesis (25–27). What distinguishes it is its facilitative synthesis approach, which recommends “discovering a ‘whole’ among a set of parts” (23). Noblit and Hare posit that it allows systematic comparisons involving translations of published work into each other, assuming that publications can be added together. The resulting interpretation from the synthesis results in additional layers of interpretations that are “metaphoric” and “not simply an aggregation of the interpretations already made in the studies [i.e., publications] being synthesized” (23, 28).

Initially, the application of the meta-ethnography method was limited to research based on ethnographical accounts; however, since then, it has been widely applied and considered suitable for interpreting diverse types of qualitative synthesis (26, 27). A recent adaptation of the method by the original developers clarifies that the method is “no longer limited to ethnographies” and has been successfully used with all forms of qualitative research (28).

The main reason we adopt the meta-ethnography approach in this review is because of one of its main strengths of allowing higher-order conceptual output that seeks to extend and enhance current conceptualizations or theories of the phenomenon of interest (25, 26); in our case, we are interested in expanding the conceptualization of implementation outcomes.

Meta-ethnography involves seven overlapping and iterative phases. *Phase 1 (getting started)* focuses on formulating the review research question. *Phase 2 (deciding what is relevant to the initial interest)* concerns being purposeful in searching and sampling publications for inclusion in the interpretive review*. Phase 3 (reading the publications)* marks the beginning of data extraction. *Phase 4 (determining how the publications are related), phase 5 (translating the publications into one another)*, and *phase 6 (synthesizing translations*) are all concerned with how to approach the data analysis and the resulting interpretive synthesis of the included publications. *Phase 7 (expressing the synthesis)* concerns the dissemination of the synthesis output beyond publication and is outside the scope of this current output. In the following three subsections, we detail the application of phases 1–6 to our review, specifically, our approach for search and sampling strategy for identifying relevant literature that aligned with our review question, the results of the included publications, and our approach to data analysis and synthesis of the included publications.

### 2.1 Searching and sampling the literature

#### 2.1.1 Initial systematic search for conceptual implementation outcomes—BeHEMoTh approach

The authors of the meta-ethnography approach recommend following a process of “specification,” which involves beginning with a purposive search that is then modified based on what the search reveals, as opposed to being overly prescriptive (28). However, this method does not specify how to sample the relevant literature and only recommends a purposive search. Thus, to identify relevant literature and as a starting point, we applied the BeHEMoTh approach to systematically search for and identify a comprehensive compilation of concepts related to implementation outcomes from diverse literature (24). The BeHEMoTh approach provides a structured way of specifying and identifying theories, models, frameworks, concepts, etc. (i.e., theoretical conceptualizations) in a systematic way.

The approach applies one of two alternative strands of systematic searching with the aims of (i) identifying any *occurrences* of theoretical conceptualizations related to a review topic or the phenomenon of interest (theoretical conceptualizations occurrence searches) and (ii) consolidating and explaining how theoretical conceptualizations have been *used* in relation to a review topic (theoretical conceptualizations use searches). For this review, we applied the first strand of searching for theoretical conceptualizations because the main aim was to identify any occurrences of theoretical conceptualizations related to implementation outcomes and source a comprehensive compilation of concepts from different publications. For this strand, Booth and Carroll (24) suggested searching in the existing databases of the review team for incidental occurrences of relevant publications (i.e., articles and book chapters) to consider for inclusion and conducting a systematic electronic database search combining search terms for the review topic with terms for theoretical conceptualizations. The BeHEMoTh approach suggests following a structure (as outlined below, which is further laid out in Table 1) to derive eligibility criteria. These structured eligibility criteria are then used to inform the design of a search strategy by covering and combining the three aspects of (i) behavior/phenomenon of interest (Be), (ii) health context (He), and (iii) models (Mo) or theory (Th). For this review, these aspects are defined as follows:

• Behavior/phenomenon of interest: implementation outcomes;
• Health context: public/not-for-profit health service delivery or management; and
• Models or theory: models, theories, frameworks, concepts, definitions, measures, and tools (theoretical conceptualizations).

**Table 1:** Eligibility criteria^a^ for searching and sampling publications for inclusion in the interpretive synthesis review.

Eligibility criteria	Inclusion	Exclusion
Behavior phenomenon of interest	Publications focusing on 1. Developing or enhancing concepts of implementation outcomes, including, for example, definitions of adoption, implementation, sustainability, and abandonment outcomes 2. The active implementation of innovations 3. Any innovation type, such as service delivery processes and models, technology and devices, programs and interventions, management practices	Publications focusing on 4. Concepts related to innovation development or knowledge/evidence use 5. Passive implementation processes 6. Outcome concepts related to effectiveness of innovations 7. Using but not developing or enhancing concepts
Health context	Publications focusing on the implementation of innovations 8. In a public or private not-for-profit context 9. In a health service delivery practice or management service, including healthcare, home, public health, or community settings (innovation implemented/delivered by health service providers or managers) 10. At any level (micro-individual, meso-organizational/setting, macro-beyond organization/system)	Publications focusing on the implementation of innovations 11. In a private for-profit context 12. In a non-health service practice context, e.g., policymaking, research, governance, and financial context 13. Only targeting/involving non-health service providers, e.g., teachers, social workers, researchers, or policymakers
Models, theories, or frameworks	14. Models, theories, frameworks, concepts, definitions, measures, and tools	15. Technical or statistical models
Types of publications	Publications published 16. As articles in scientific journals, books, and book chapters 17. In the English language 18. Of any research design such as empirical experimental or observational studies, conceptual publications, reviews 19. As final/full publications including original research, debate, editorials, and research protocols	Publications published 20. As conference abstracts, 21. In gray literature and dissertations

^a^ Eligibility criteria used for guiding both initial systematic identification of potential publications for inclusion (using the BeHEMoTh approach) and subsequent purposive searching and sampling by building on the initial search results.

Following the BeHEMoTh approach, we designed an initial search strategy based on our eligibility criteria. The following electronic databases were systematically searched: Medline, Embase, CINAHL, PsycInfo, Global Health, HMIC, Business Source Complete, and Social Policy and Practice. These databases cover multiple disciplines, including medical and health sciences, implementation science, and social sciences, including organization and management research. As our review aimed to identify conceptualizations of implementation outcomes (which might be presented as or part of theories, models, frameworks, or other standalone conceptualizations) published in peer-reviewed journals or books, we did not include gray literature. The search strategy was adjusted to the different databases, combining subject headings and free-text terms for behavior/phenomenon of interest (implementation outcomes) AND health service context AND theoretical conceptualizations. For the behavior/phenomenon of interest, we also searched for different descriptors of implementation outcomes, e.g., level, strength, intensity, depth, or extent. Searches were limited to articles and book chapters published in English from 2004 onward. We chose this year as it marks the seminal publication by Greenhalgh et al., which is widely recognized as a foundational work in the field of implementation science in healthcare (29).

The initial search and sampling of publications for inclusion, using the BeHEMoTh approach, was carried out in December 2020. Citations were managed using EndNote X9 and Rayyan (30). Citations were independently double-screened by three reviewers, with one reviewer screening 100% (AZ and ZB) and the other two reviewers (AZ and CS) screening 50% each for both titles/abstracts and full texts. Disagreements were resolved through discussion and consensus within the review team. As the BeHEMoTh approach only offers a starting point for identifying potential theoretical or conceptual publications to consider for inclusion, we carried out additional purposive sampling based on the results of this initial systematic search, as detailed below.

#### 2.1.2 Subsequent purposive search and sampling

After the initial strategy of systematically searching for conceptual implementation outcomes for assessing implementation efforts using the BeHEMoTh approach, the second strategy we employed involved the following techniques to purposively identify relevant publications for inclusion: we included publications by hand-searching reference lists of included publications, reviewing key textbooks in the fields of implementation science and innovation research, and citation tracking of included publications between January 2021 and January 2022. In cases where an included publication was found to further develop or enhance the conceptualization of an outcome, we ensured to source the original publication to consider it for inclusion. After exhausting relevant citation sources connected to already identified publications, we proceeded with purposive searching for publications, e.g., using Google Scholar, on specific outcome concepts we identified during data analysis to fill gaps in the synthesis, enrich the analysis, and include newer publications. These searches were carried out iteratively throughout the later stages of our analysis between February 2022 and September 2023.

To examine publications identified during the subsequent purposive search and sampling for inclusion, one reviewer screened all publications to determine whether they (i) developed implementation outcome concepts/contributed to their theoretical conceptualizations or (ii) mentioned/applied but not (further) developed theoretical conceptualizations of outcomes. Publications in the first group were included in the synthesis; publications in the second group were excluded. For the excluded group, key original publications contributing to theoretical conceptualizations mentioned or applied in these publications were retrieved and included in the synthesis. The excluded group contained, for example, empirical articles/chapters that used theoretical conceptualizations in question to inform data analysis but did not contribute to their further development.

#### 2.1.3 Data extraction

Data extraction activities (phase 3 of the meta-ethnography approach) entail reading the identified publications closely. It overlaps with the other phases, as iterative close reading of the included publications is undertaken throughout the review process. Second-order concepts, which Noblit and Hare termed “interpretative metaphors” (23)—in our case, implementation outcomes—were extracted from all publications considered eligible for inclusion. In this review, we extracted data on the following aspects of the second-order conceptual implementation outcomes described in each publication: outcome labels, outcome definitions, and outcome characteristics/attributes. In total, we extracted 55 individual implementation outcome labels. We excluded further publications if they did not include sufficient data on outcome aspects. In addition, we also extracted the following data on publication characteristics: first author's name; publication year and title; publication aim/research question; design/method; discipline (e.g., medical and health sciences, implementation science); application field (e.g., wider healthcare system, public/community health); and country, region, and theory name (where applicable). Data extraction was conducted by two members of the research team (AZ and ZB).

### 2.2 Included publications

**Figure 1:**
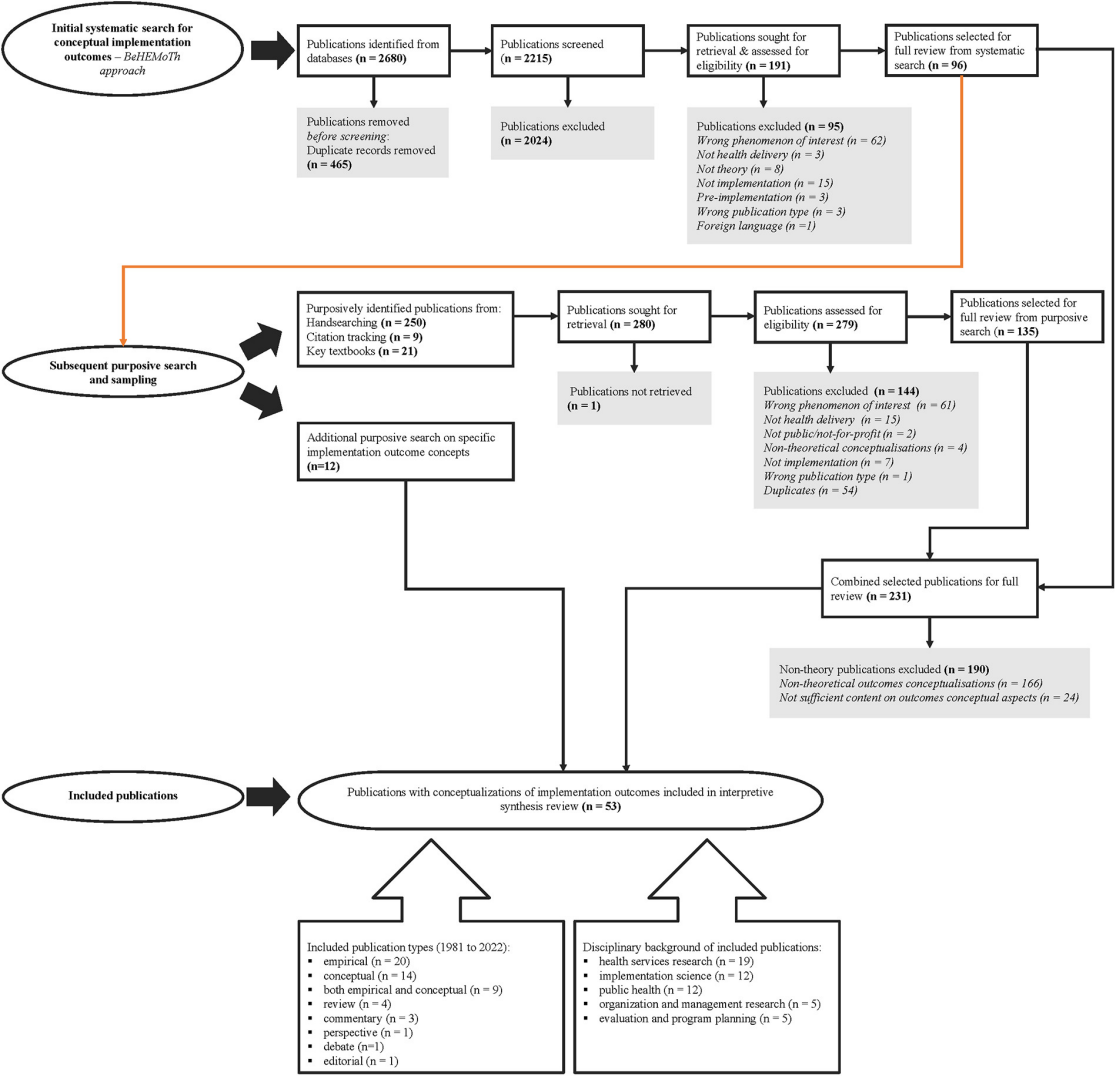
Flowchart of publications’ search and selection processes. BeHEMoTh, Behaviour or phenomenon of interest, Health context, Exclusions, Models, and Theories.

The search and sampling of the literature resulted in the inclusion of 53 publications. Of these 53 included publications, 41 were identified via the initial systematic search using the BeHEMoTh approach across eight electronic databases, along with purposive sampling via hand-searching, citation tracking of included publications, and review of key textbooks. The other 12 included publications were identified via an additional purposive search on specific implementation outcomes. Figure 1 presents an overview of the process we followed in sourcing and identifying potential publications for inclusion and a summary of key characteristics of the included publications.

The publication year of the 53 included publications ranged from 1981 to 2022. Most of the publications were theoretically relevant empirical publications (*n* = 20), followed by conceptual publications (*n* = 14), with the rest being publications that were both empirical and conceptual (*n* = 9), reviews (*n* = 4), commentaries (*n* = 3), a perspective ( *n* = 1), a debate piece (*n* = 1), and an editorial (*n* = 1). The disciplinary backgrounds of the included publications spanned a range of fields, including health services research (*n* = 19), implementation science (*n* = 12), public health (*n* = 12), organization and management research (*n* = 5), and evaluation and program planning (*n* = 5). The indicated application field of the included publications commonly included evaluation of (evidence-based) innovations (*n* = 42), organizational change (*n* = 5), quality improvement (*n* = 5), and reporting guidelines (*n* = 1).

### 2.3 Data analysis and synthesis

As mentioned in the above overview of the methods, meta-ethnography suggests the synthesis of interpretations extracted from the included publications (e.g., conceptual implementation outcomes and the associated explanations) in a manner that generates additional layers of interpretation during a process of translating publications into one another. To achieve this, Noblit and Hare suggested three forms of translation: *reciprocal translational analysis (RTA)*—applicable when the extracted theoretical conceptualizations of the phenomena of interest (in our case, implementation outcomes) are directly translatable into one another; *line of arguments (LOA)*—applicable in cases where there is overlap, but a closer comparison reveals that the extracted concepts address different aspects of a larger explanation; and *refutational synthesis (RS)*—applicable when there are some contradictions between extracted concepts, i.e., they do not add up (23, 28). For RS, a further step is to substantively consider the implied relationships between competing explanations of extracted concepts and include them as part of the synthesis (23). For example, we applied RTA in cases where two extracted outcomes were deemed translatable, thus conveying the same conceptual explanation—“implementation” and “implementation quality.” We applied LOA, for example, in the interpretive synthesis of “institutionalization” and “sustainability” outcomes, which overlap in their conceptualizations but delineate different aspects of what it means to integrate an innovation. We applied the RS translation approach to distinguish between the extracted outcomes—“penetration” and “reach” because even though, at the surface level, the conceptual definition of these two concepts implied some degree of overlap, their detailed conceptual explanations did not sufficiently satisfy placing them under the same category.

We began with a tentative analysis of the extracted second-order concepts of implementation outcomes based on similarities in their conceptual meanings using RTA and LOA translation approaches as appropriate as analytical lenses to examine their relatedness and translatability. The application of RS is more of a consequential step that emerges from applying the other two forms of translation (RTA and LOA). Applying the latter two indicates the necessity to substantively consider the implied relationship between competing conceptual explanations of the outcomes. Thus, implied refutations between the conceptual meanings of extracted outcomes led us to formulate distinct categories and establish boundary conditions between categories. We tentatively categorized extracted outcomes that were either translatable or exhibited overlapping conceptualizations into emergent third-order constructs, which we eventually labeled as either *modes* or *attributes*. We then conducted a more detailed inspection of conceptual definitions and explanations of the extracted outcomes to produce synthesized, broader conceptual definitions of the individual outcomes clustered within the modes and attributes. We report the findings and analysis outputs of the individual modes and attributes in the following section. Table 2 lists key terms we used in this review and their definitions to serve as a reference point.

**Table 2:** Terms and definitions.

Term	Definition
Implementation outcome(s)	Indicators of implementation effectiveness of innovations by pointing to the consequences or effects of implementation efforts for innovations
Mode(s)	Distinguish focal implementation outcomes of the implementation process and are grouped based on the condition of the overall implementation process
Attribute(s)	Distinguish implementation outcomes that characterize the thoroughness of the overall implementation process; offer a re-conceptualization of applicable existing implementation outcomes that act as the features of the modes of implementation outcomes
Concept	“A general idea or notion, a universal; a mental representation of the essential or typical properties of something, considered without regard to the peculiar properties of any specific instance or example” (31)
Reciprocal Translational Analysis (RTA)	Part of the meta-ethnography analytical approach; applied when extracted concepts (i.e., prior conceptualizations) of the phenomena of interest are directly translatable into one another (23)
Line of Arguments (LOA)	Part of the meta-ethnography analytical approach; applied when there is an overlap between concepts of the phenomena of interest, but a closer comparison reveals that concepts address different aspects of a larger explanation (23)
Refutational Synthesis (RS)	Part of the meta-ethnography analytical approach; applied when there are contradictions between extracted concepts of the phenomena of interest (23)
Operationalization	When a concept is described to enable practical and measurable observations (e.g., development of implementation outcomes instruments to define operational measures derived from concepts of implementation outcomes)
Implementation efforts	Implementation efforts encompass implementation components that are typically understood to form implementation strategies deployed to implement change, i.e., implementation strategies are defined as “systematic processes, methods, techniques, activities, and resources that support the adoption, integration, and sustainment of evidence-based interventions into usual settings” (102)

## 3 Findings and analysis: integrated re-conceptualization of implementation outcomes

Implementation outcomes are understood to function as indicators of the implementation effectiveness of innovations. After reviewing their existing conceptualizations, we mapped and consolidated synthesized interpretations to extend established conceptualizations of implementation outcomes. We conceptualized two main components of implementation outcomes as *modes* and *attributes* by considering the relationships between and across the outcomes and their roles in the overall process of implementation efforts. Based on the analysis of the synthesized implementation outcomes, modes refer to distinct conditions of the implementation process. Modes specify focal implementation outcomes of the overall implementation process. Attributes distinguish implementation outcomes that characterize the thoroughness of the overall implementation process. Attributes classify individual implementation outcomes to indicate the extent of depth, breadth, progression, and adaptation of implementation efforts and the extent of de-implementation (e.g., the extent of unlearning) associated with the modes of implementation outcomes. While the modes refer to implementation outcomes at the core of implementation efforts, the relevance of evaluating outcomes linked to the attributes will vary depending on the circumstances and requirements of implementation efforts.

The modes comprise *engagement*, *active implementation*, and *integration*. The attributes associated with the modes are *implementation depth*, *implementation breadth*, *implementation pace*, *implementation adaptation*, and *de-implementation*. In the following subsections, we discuss the synthesized extracted implementation outcomes concerning each mode and attribute and how they add to the conceptual clarity and/or advance re-conceptualization of implementation outcomes.

### 3.1 Modes of implementation outcomes

#### 3.1.1 Engagement mode

*Engagement mode* explains the perceived fit, capacity, usability, and intention to adopt an innovation or a passive loss of engagement (i.e., failure of engagement, which leads to non-adoption) and adoption rate within or across settings. When we examined the conceptual definitions of the implementation outcomes related to the “adoption” process of the overall implementation process, the lines-of-argument analytical approach (i.e., outcomes overlap but delineate different aspects of the adoption process) was most appropriate to compare conceptual definitions. However, we deemed creating the term “engagement” to label this mode of implementation outcomes, rather than adopting the term adoption, to be able to capture the holistic meaning conveyed by all the outcomes mapped under this mode (Table 3).

**Table 3:** Engagement mode and associated implementation outcomes.

Modes of implementation outcomes—engagement	Extracted implementation outcomes	Definitions of extracted implementation outcomes (and first author's name and publication year)
Synthesized interpretation of extracted outcomes: Line of Arguments (LOA): All outcomes mapped to the engagement mode of implementation outcomes overlap, as they relate to the adoption process of the overall implementation process. However, they delineate different aspects that explain early indications of the innovation adoption process, including the assessment of perceived fit, capacity, usability, and intention to use an innovation, as well as the passive loss of engagement (i.e., failure of engagement, which leads to non-adoption) and the rate of adoption.	Readiness level	Commitment and ability to implement a new service; readiness as an essential precursor of early implementation outcomes; readiness is assessed using the “R = MC^2^” heuristic, defined as the interplay of three components: *motivation* (i.e., perceived incentives and disincentives that contribute to the desirability of an innovation), *general capacity* (i.e., conditions related to how well an organization is functioning), and *innovation-specific capacity* (i.e., conditions needed to implement a specific innovation) (Livet 2022) (20)
	Adoption	Number, proportion, and representativeness of settings, practices, plans, and intervention agents that adopt an intervention (Glasgow 1999) (12) Intention, initial decision, or action to try or employ an innovation or evidence-based practice (Proctor 2011) (10)
	Innovation—decision process: adoption or rejection	Engaging in activities that lead to a choice to adopt (a decision to make full use of an innovation as the best course of action available) or reject the innovation (a decision not to adopt an innovation) (Rogers 2003) (32)
	Later adoption	Adoption of the innovation after a previous decision to reject it (Rogers 2003) (32)
	Rate of adoption	The relative speed with which an innovation is adopted by members of a social system; it is generally measured as the number of individuals who adopt a new idea in a specified period such as a year (Rogers 2003) (32)
	Acceptability	The perception among implementation stakeholders that a given treatment, service, practice, or innovation is agreeable, palatable, or satisfactory (Proctor 2011) (10) The extent to which people delivering or receiving a healthcare intervention consider it appropriate, based on anticipated or experiential cognitive and emotional responses to the intervention (Sekhon 2017) (33)
	Appropriateness	Perceived fit, relevance, or compatibility of the innovation or evidence-based practice for a given practice setting, provider, or consumer; and/or perceived fit of the innovation to address a particular issue or problem (Proctor 2011) (10)
	Feasibility	The extent to which a new treatment or an innovation can be successfully used or carried out within a given agency or setting (Proctor 2011) (10)
	Non-adoption	Passive rejection, which consists of never really considering the use of the innovation (Rogers 2003) (32)

As shown in Table 3, the outcomes mapped to this mode represent the initial or later decision to adopt an innovation. These include “adoption” (10); understanding “proportion” of adopting units/agents or “rate of adoption” (12, 32); indication of “readiness level” (20); perceptual outcomes, such as—“acceptability” (10, 33) and “appropriateness” and “feasibility” (10), which are known to predict adoption (15); and “non-adoption”—defined as a “passive rejection that consists of never really considering the use of the innovation” (32). The engagement mode of implementation outcomes thus integrates outcomes for evaluating the initial commitment or promise to try an innovation, the related perceptions and activities that lead to this commitment, or the unintended/undesired consequence of the adoption process. The engagement mode also includes outcomes that indicate the proportion and rate of the commitment by the engaged units or agents.

#### 3.1.2 Active implementation mode

The *active implementation mode* represents implementation outcomes that indicate the use (or non-use) of an innovation, along with considering the level of fidelity and the associated implementation cost. This mode typically follows the engagement mode, as a natural sequential step of the implementation efforts is to proceed with active implementation of the innovation once a sufficient level of engagement has been established. The cluster of implementation outcomes we synthesized under the active implementation mode conveys a general or specific conceptual meaning (Table 4). The implementation outcome labeled as “implementation” (7, 32) conveys a general or broader meaning, referring to the extent to which an innovation is being delivered, put into use, and operationalized to signal the transition to the active implementation mode (7, 32).

**Table 4:** Active implementation mode and associated implementation outcomes.

Modes of implementation outcomes—active implementation	Extracted implementation outcomes	Definitions of extracted implementation outcomes (first author's name and publication year)
Synthesized interpretation of extracted outcomes: Line of Arguments (LOA): Implementation outcomes mapped to active implementation mode overlap but delineate different aspects regarding *implementation* (i.e., putting innovation to use), *fidelity*, and *cost* of implementation efforts or *implementation failure*.	Implementation	The extent to which the innovation is in place or being delivered (Damschroder 2022) (7) Implementation occurs when an individual (or other decision-making unit) puts an innovation into use (Rogers 2003) (32)
	Fidelity	The degree to which a treatment is implemented as intended (Moncher 1991) (34) *Implementation*: The extent to which the intervention is implemented as intended in the real world at the organizational level (Glasgow 1999) (12) The degree to which an intervention or program is delivered as intended (Carroll 2007) (35) Organizational members’ level of commitment to using the distinct components that make up an intervention as they were (Keith 2010) (36) The degree to which an intervention was implemented as it was prescribed in the original protocol or as it was intended by the program developers (Proctor 2011) (10) *Quality implementation:* Putting an innovation into practice in such a way that it meets the necessary standards to achieve the innovation's desired outcomes (Meyers 2012) (37)
	Cost	The cost impact of an implementation effort (Proctor et al., 2011) (10) Cost of implementation (time, cost, resources) (Glasgow 1999) (12)
	Implementation failure	When a planned process or set of strategies is not properly put into practice (Katz 2013) (38) A new project or strategy that was formulated and not implemented or one that was implemented but with poor results (Decker 2012) (39)

In contrast, outcomes that convey a specific meaning of active implementation may overlap, but a comparison reveals that they capture relevant and distinct aspects that need to be considered when innovations are put into use. These outcomes are “fidelity” (10, 12, 34–37), “cost” (10, 12), and “implementation failure” (38, 39). The specific conceptual meanings of these outcomes indicate the degree to which the innovation was delivered as intended (fidelity) (10, 12, 34–37), taking stock of implementation cost (cost) (10, 12), and improper execution of implementation plans/strategies or poor results after active use of the innovation, which leads to reverting to the pre-implementation state (implementation failure) (38, 39). Some authors use the labels “implementation” (12) and “quality implementation” (37) to refer to a conceptual definition that is consistent with the conceptual meaning of fidelity. Thus, we translated these two individual outcomes as synonymous with fidelity, as similarly defined by the other authors (10, 34–36). Taken together, we propose that the active implementation mode represents a conceptual reduction of outcomes under this cluster, which serves as a necessary transitional mode between the engagement mode and *integration mode*, as discussed below, that may lead to routinized and enduring desired consequences.

#### 3.1.3 Integration mode

The *integration mode* reflects implementation outcomes that arise when an innovation is embedded—specifically, its structural and procedural integration as enacted in everyday practice over a defined (or extended) period. This mode also nuances assessing the likelihood of continued integration of the innovation. Conversely, it also points to a failure to integrate an innovation.

The outcomes mapped to the integration mode (Table 5) are typically all observable, in more tangible ways, or have more resonance following the effective active implementation of an innovation. Thus, they overlap in this way in their conceptual meanings. However, there are some distinctions that foreground five main characteristics of implementation outcomes associated with the integration mode, which are (i) integration in relation to an organization's structural and procedural conditions (i.e., “institutionalization”) (32, 40–42); (ii) integration in relation to conditions of everyday practice [i.e., “routinization” (42, 43) or *“*normalization” (44, 45)]; (iii) actual integration in relation to a specified or longer term timeframe [i.e., “maintenance” (12, 46), “sustainability” (10, 39, 42, 47–57), or “sustainment” (7, 58)]; (iv) potential integration in relation to capacity for continued delivery [i.e., “capacity for sustainability” (59), “sustainability planning” (47), or “sustainability” (7, 57, 58)]; and (v) failure to integrate an innovation [“sustainability failure” (50)].

**Table 5:** Integration mode and associated implementation outcomes.

Modes of implementation outcomes—integration	Extracted implementation outcomes	Definitions of extracted implementation outcomes (first author's name and publication year)
Synthesized interpretation of extracted outcomes Line of Arguments (LOA): Outcomes mapped to integration mode of implementation outcomes overlap in that they all indicate consequences of or capacity for integrating an innovation but delineate different aspects that reveal what it means to integrate an innovation structurally/procedurally (i.e., *institutionalization*) and in everyday practice (*routinization/normalization*) over a specified or extended time period (actual *maintenance* and *sustainability/sustainment*), as well as likelihood for sustainability or failure to integrate an innovation (*sustainability failure*).	Institutionalization	Successful institutionalization means programs “settle” into their host organizations as integrated components; factors associated with it include standard operating routines, clusters of critical precursor conditions, and fit between the program and the organization's mission and core operations (Goodman 1989) (40) Attainment of long-term viability and integration of programs within organizations; final stage in an innovation diffusion process (Goodman 1989) (41) A point is reached at which the new idea becomes institutionalized as a regularized part of an adopter's ongoing operations; the innovation loses its distinctive quality as the separate identity of the new idea disappears (Rogers 2003) (32) Gradual adaptation of the organizational context, including structures and processes, to the new work practice (Slaghuis 2011) (42)
	Routinization	Becoming part of “standard practice”; three degrees are involved: marginally, moderately, and highly routinized (Yin 1981) (43) Through the development of organizational routines, a new work method becomes part of everyday activities (Slaghuis 2011) (42)
	Normalization	Technology becomes one of several means by which services can be delivered; it ceases to be a special application and instead becomes one of the normal arms of clinical practice (May 2003) (44) Practices become routinely embedded in social contexts as a result of people working, individually and collectively, to enact them (May 2009) (45)
	Maintenance	The extent to which innovations become a relatively stable, enduring part of the behavioral repertoire of an individual (or organization or community); the extent to which a program is sustained over time (Glasgow 1999) (12) Assessment of short-term maintenance of innovations post-implementation, an indicator of early maintenance of innovation (Shelton 2020) (46)
	Sustainability (actual continued sustainment)	Program continuation (permanence, time) without limiting its manifestations to any particular form (Shediac-Rizkallah 1998) (47) Routinization within organizations; three degrees are involved: (1) weak sustainability—absence of routine; (2) medium sustainability—presence of non-standard routines; and (3) strong sustainability—presence of standardized routines (Pluye 2004) (48) New working methods, performance goals, and improvement trajectories are maintained for a period appropriate to a given context (Buchanan 2005) (49) Continued use of core elements of an intervention and persistent gains in performance as a result of those interventions (Bowman 2008) (50) When new ways of working and improved outcomes become the norm; change has become an integrated or mainstream way of working rather than something “added on”; holding the gains and evolving as required, not going back (Maher et al., 2010) (51) An implemented change will continue to be in place or will have been improved upon 6 months later (Molfenter 2011) (52) The extent to which a newly implemented treatment is maintained or institutionalized within a service setting's ongoing, stable operations (Proctor 2011) (10) A new work method becomes part of everyday activities; sustainability is conceptualized with two dimensions: routinization and institutionalization (Slaghuis 2011) (42) The continued use of program components and activities for the continued achievement of desirable program and population outcomes; continued use of program components and activities beyond their initial funding period; and continuation of desired intended outcomes (Scheirer 2011) (53). Continuation or integration of a new practice within an organization whereby it has become a routine part of care delivery and continues to deliver desired outcomes (Doyle 2013) (54) *Sustainment:* Continued use of an intervention within practice (Chambers 2013) (58) Longer-term endurance of an innovation, defined by three characteristics: benefits, routinization or institutionalization, and development; it implies the stability of ingrained change and the dynamism of continuing change (Fleiszer 2015) (55). Maintained long term through adaptation to context over time; persisted long term (Greenhalgh 2017) (56) Continued delivery of the innovation and continued receipt of benefits (Urquhart 2020) (57) *Sustainment*: The extent the innovation is in place or being delivered long-term (Damschroder 2022) (7)
	Capacity for sustainability	*Capacity for sustainability:* Assessing the characteristics of a program, its parent organization, and its place in the larger service system context that leads to program sustainability (Schell 2013) (59) *Sustainability planning:* Understanding the conditions under which programs will most likely continue. Three major factors are potential influencers on sustainability: (1) project design and implementation; (2) factors within the organizational setting; (3) factors in the broader community environment (Shediac-Rizkallah 1998) (47) *Sustainability:* The extent to which an evidence-based intervention can deliver its intended benefits over an extended period of time after external support from the donor agency is terminated (Chambers 2013) (58) *Sustainability:* Likelihood that the innovation will be put in place or delivered over the long term (Damschroder 2022) (7) *Sustainability:* Continued capacity to deliver innovation (Urquhart 2020) (57)
	Sustainability failure	Performance is returning to baseline, and in the worst case, dropping below it; sustainability failure is associated with a lack of system thinking, that is, to capitalize on gains made during the active phase (Bowman 2008) (50)

Hence, the first two aspects of the integration mode refer to features of an organization's context. The first aspect, institutionalization, is more commonly associated with the innovation becoming part of the organization's procedural practices, core competencies, and the values of organizational members (42), as well as being regularized within an organization's ongoing operations (32, 40, 41). The second aspect, routinization and normalization, refers to an innovation becoming part of the organization's everyday activities (42–45). The third aspect of integration, which we associate with maintenance and sustainability/sustainment outcomes, conveys a comprehensive conceptual meaning that not only refers to the innovation being officially in place (institutionalization) and use in daily activities (routinization/normalization) but also to the extent the innovation is integrated for a specified term (short-term maintenance) (12, 46) or a longer-term (long-term sustainability) (7, 10, 39, 42, 47–58). The fourth aspect conceptually delineates what it means to prepare for and determine continued capacity to integrate an innovation. The final aspect of the integration mode refers to a failure to embed and maintain gains achieved during the active implementation mode (sustainability failure) (50).

Notably, in the included publications, the concept of “sustainability” is used interchangeably to refer to either an outcome that indicates the actual continued delivery of an innovation or the potential for continued delivery (i.e., capacity for sustainability). Of the 16 included publications in our review that conceptualize what it means to evaluate outcomes related to sustaining an innovation, 14 use the “sustainability” concept to explain the actual continued delivery of an innovation (7, 10, 42, 47–52, 54–58), whereas only two publications make a conceptual distinction between “sustainability” and “sustainment”—reserving sustainability for assessing capacity to sustain the innovation and sustainment for the actual continued use of the innovation (7, 58). In addition, in 5 of the 16 publications, authors offer conceptual clarity on assessing capacity for sustainability, albeit using varied concept labels (Table 5) (7, 47, 57–59).

### 3.2 Attributes associated with the modes of implementation outcomes

#### 3.2.1 Implementation depth

*Implementation depth* is defined as the inward internalization of an innovation within and/or across specific organizational units and indicates the innovation's temporal progression and degree of implementation in three dimensions: (i) the extent of use within each part of an organization (i.e., quantity and quality of implementation efforts in each part); (ii) the reach of the innovation in all parts of an organization (i.e., organizational immersion as a whole); and (iii) the amount of time taken to achieve each of these two dimensions of depth (i.e., depth of implementation against a timeline). The extracted implementation outcomes that convey this integrated conceptual meaning have definitions that overlap but emphasize different aspects of implementation depth.

With regard to the terminology used by authors (Table 6), the outcomes that explain both the depth of implementation efforts in each part and the whole organizational immersion are referred to in the publications included as “assimilation” (60) and “penetration” (10). Outcomes such as “depth” (61), “level” (62, 63), and “effectiveness” (64) are also used to refer both to the part and the whole and include time dimension in their conceptualizations. In contrast, outcomes such as “intensity” or “strength” (65, 66), “dose” (67), “completion” (68), “infusion” (60, 69), and “scale-up” (56) mainly focus on explaining the depth of implementation at the organizational level or the whole.

**Table 6:** Depth**—**as an attribute of the modes of implementation outcomes.

Attributes of the modes—depth	Extracted implementation outcomes	Definitions of extracted implementation outcomes (first author's name and publication year)
Synthesized interpretation of extracted outcomes: Line of Arguments (LOA): The nine different extracted implementation outcomes mapped to depth are reconceptualized as part of the attributes associated with modes of implementation outcomes. The individual outcomes overlap to characterize the inward internalization of innovations within and/or across organizational units. However, they do so from three different angles that explain the degree of implementation efforts within individual organizational units, the degree of organizational immersion as a whole, and against a specified timeline. Assessing implementation depth reveals both the quantity and quality of change effort at the unit and wider collective levels.	Assimilation stage	How deeply the innovation penetrates the adopting unit (e.g., the company, division, or workgroup); the degree of implementation within the adopting unit as a whole; assimilation may be divided into two subconstructs: breadth of use (number of adopters within a firm, i.e., internal diffusion); and depth of use (how extensively the innovation is used and its level of impact within the organization) (Gallivan 2001) (60)
	Penetration	The integration or saturation of practice within a service setting and its subsystems (Proctor 2011) (10)
	Depth	Length of time the hospital had been involved in the program, the total number of program elements and volume of activities, the extent of the link between the program and patient satisfaction, and the time and extent of organizational immersion in the program (Carman 1996) (61)
	Level	*Level or depth*: Program penetration in the organization (did it reach all parts?), how “deeply” it was applied in each part and for how long it was applied; the extent to which the program was actually carried out; was the intervention implemented fully, in all areas and to the required “depth,” and for how long (Øvretveit 2002) (63) *Level*: Low, medium, and high, the extent to which innovation is implemented (Liang 2016) (62) *Effectiveness*: Facilities with program components actually implemented and participation rates in the following year; subcategories high, low, and in transition (Damschroder 2013) (64)
	Intensity	Quantity and depth of the intervention change activities (Pearson 2005) (66) *Intensity or strength*: Amount of input to or activity to support the implementation of a program (Hargreaves 2016) (65)
	Dose	Quantity of intervention implemented (Moore et al., 2015) (67)
	Completion	Completion of implementation activities, the length of time taken to complete activities, and the proportion of activities completed (Chamberlain 2011) (68)
	Infusion	The innovation penetrates the organization; increased organizational effectiveness is obtained by using the application in a more comprehensive and integrated manner to support higher-level aspects of work (Gallivan 2001) (60) The extent to which an innovation is used completely and effectively, perhaps more at the individual level (Wainwright 2007) (69)
	Scale-up	Fully mainstreamed and part of business as usual locally (Greenhalgh 2017) (56)

Outcomes mapped to implementation depth are defined as ratings that indicate implementation effectiveness by assessing facilities that actually implemented an innovation's components (64); completion of implementation activities and proportion of activities completed (68); integration of practices within a setting and its subsystems (10); the reach across all parts of an organization, the depth of application within each part of an organization, and the extent to which an innovation is implemented (63); the penetration of the innovation into an organization (10, 60, 63); and the timeframe and extent of organizational immersion (61). As an attribute of the modes, the evaluation of implementation depth offers insight into the extent of comprehensiveness and saturation of implementation efforts across all parts of the organization over time.

#### 3.2.2 Implementation breadth

We interpret *implementation breadth* as a second attribute of the modes of implementation outcomes, defined as the extent of outward distribution of innovations across organizations, sites, settings, or populations, encompassing both spontaneous and planned efforts coupled with equitable distribution. This synthesized conceptual meaning of implementation breadth is based on the similarities we observed across related implementation outcomes extracted from the included publications. However, some differences foreground three main aspects of implementation breadth: breadth at the organization/site/setting level, breadth at both the organization/site/setting level and population level, and breadth at the population level.

The extracted implementation outcomes labeled as “breadth,” referring to the reach of an innovation across all parts of an organization or health systems (63), and “spread,” denoting the transfer of an innovation to new settings (56), explicitly capture the evaluation of breadth at the organizational/site/setting level. The extracted outcomes labeled as “diffusion,” which refers to the dissemination of an innovation beyond the original organization/site/setting/population (70), and “intentional spread/uptake,” defined as systematic efforts to extend effective innovations to wider populations or specific services (71), explicitly reflect breadth both at the organization/site/setting level and the population level.

In contrast, the extracted outcomes labeled as “reach,” referring to the proportion of the target population participating in intervention (12), “scale/scalability,” indicating expansion of an innovation to reach a greater proportion of the eligible population (72), “scaling-up,” denoting efforts to increase the impact of innovations to benefit more people (73), and Roger's conceptualization of “diffusion,” indicating communication of an innovation among the members of a system (32), all foreground assessment of breadth at the population level in their conceptual meanings. The final outcome mapped under implementation breadth—“equitable implementation”—complements all the other outcomes in this category by pointing to the assessment of equity representativeness in the implementation of innovations across different population subgroups and settings (46) (Table 7).

**Table 7:** Breadth**—**as an attribute of the modes of implementation outcomes.

Attributes of the modes—breadth	Extracted implementation outcomes	Definitions of extracted implementation outcomes (first author's name and publication year)
Synthesized interpretation of extracted outcomes: Line of Arguments (LOA): Implementation outcomes mapped to breadth overlap in their conceptual definitions, revealing breadth as the outward distribution of innovations. However, they delineate different adopter levels to explain implementation breadth is not limited to the extent of breadth at the organizational/site/setting level (as the provider of the innovation) but the extent to which it reaches or is received by the eligible population or beneficiary at the individual level. Further distinctions are also drawn up by some outcomes about the intentionality and assumptions that underlie the approach to spread efforts. Breadth may also include evaluating the extent of equitable representativeness in innovation implementation across different population subgroups and settings.	Breadth	How “broadly” did the program penetrate the organization (did it reach all parts?) or across health systems (Øvretveit 2002) (63)
	Diffusion	Passive diffusion or active dissemination of successful programs beyond the original population; additional organizations adopt and deliver the program, so the program life cycle starts again (Bopp 2013) (70) Innovation is communicated among the members of a social system and includes both the planned and spontaneous spread of new ideas (Rogers 2003) (32)
	Reach	The proportion of the target population that participated in the intervention (at the individual level, e.g., patient or employee) (Glasgow 1999) (12)
	Scale/scalability	The expansion of a health intervention under real-world conditions to reach a greater proportion of the eligible population while retaining effectiveness (Milat 2013) (72)
	Spread	Transfer to new settings (Greenhalgh 2017) (56)
	Scaling-up	Efforts to magnify the impact of innovations successfully tested in pilot or experimental projects so as to benefit more people (Simmons 2007) (73)
	Intentional spread/uptake	Systematic efforts to bring effective innovations to wider populations or specific services; there are three approaches that distinguish how changes are spread: - *hierarchical control:* spread as being a directed, controlled approach led by “implementers,” - *participatory adaptation:* more decentralized and participatory but retains accountability and a belief in rational planning, and - *facilitated evolution:* emphasizes creating conditions under which “take-up sites” are able to find, adapt, and develop practices and models of care that address the challenges they face (Øvretveit 2011) (71)
	Equitable implementation	Equity in capacity, resources, adoption, execution, delivery, and sustainment of implementation efforts to achieve representativeness in the depth and breadth of innovations across different types of patient/population subgroups of focus (e.g., by race/ethnicity, age, disability, insurance status, literacy level, social determinants of health), and settings (e.g., urban/rural, lower vs. higher resource settings) (Shelton 2020) (46)

When considered together, the interpretive synthesis of outcomes mapped to implementation breadth reveals that breadth can be observed at the organization/site/setting level or the population level. Furthermore, the outcome “intentional spread/uptake” further encapsulates the intentionality behind the implementation breadth of innovations (i.e., whether it is linked to passive/spontaneous or active/planned efforts). In cases where breadth is linked to active spread efforts, these three types of spread approaches—hierarchical control, participatory adaptation, and facilitated evolution—may explain assumptions that underlie active/planned spread efforts (71).

The evaluation of breadth may be appropriate during the engagement and active implementation modes of implementation outcomes, as authors conceptualizing the synthesized outcomes of breadth refer to bringing, expanding, or further adopting an innovation that has been successfully tested in a pilot project or at a small scale to new adopters. This suggests it is possible to conceive evaluating the breadth of the engagement mode to gauge the early successes of a spread effort. Likewise, in the synthesized conceptual meanings of breadth, authors also refer to the number of adopters who used, participated in, or received the innovation or the extent to which the innovation has reached, thus again suggesting the pertinence of evaluating the breadth of active implementation.

Something worth noting is some of the ambiguity we encountered in the labeling of implementation outcomes or their conceptual definitions provided by authors. An example of the first type is the use of similar outcome labels that refer to distinct conceptual meanings, such as “scale-up,” “scaling-up,” and “scale/scalability.” In our synthesis, we mapped “scale-up” under implementation depth, as Greenhalgh and colleagues conceptualized “scale-up” as a limited local spread within an organization (56). In contrast, we mapped “scaling-up” and “scale/scalability” under implementation breadth because these outcome labels were used to describe active or intentional spread where efforts are made to expand innovations beyond the original site (72, 73). However, as also acknowledged by other authors (71), we noted that the outcome concepts “scale” and “spread” are used interchangeably in the literature concerning the evaluation of implementation efforts.

The second type of ambiguity we encountered was the conceptual definitions provided by Øvretveit and Gustafson (63) for the extracted outcome “breadth,” where it is used to imply both internal (i.e., reach within all parts of the organization) and external (i.e., reach across organizations) breadths of implementation. However, we limited mapping breadth as an extracted outcome under the derived attribute—breadth—because the other extracted conceptual outcomes—“level/depth”—by the same authors, which we mapped under implementation depth, explain the conceptual meaning of internal breadth (i.e., depth).

#### 3.2.3 Implementation pace

*Implementation pace*, the third attribute of the modes of implementation outcomes, is defined as the rate of progression of the overall implementation process measured against predefined milestones or the pace at which key milestones are achieved within the implementation modes (74). We only identified one publication by Proctor and colleagues that develops a conceptualization of how to assess “pace” in implementation efforts, included as part of a broader concept—Framework to Assess Speed of Translation (FAST) (74). In this framework, measuring pace is conceptualized to include the time from an innovation's availability to adoption, the time required to train providers to deliver innovations with fidelity, and the time to scale-up within an organizational unit or a setting (74) (Table 8).

**Table 8:** Pace**—**as an attribute of the modes of implementation outcomes.

Attributes of the modes—pace	Extracted implementation outcomes	Definitions of extracted implementation outcomes (first author's name and publication year)
Synthesized interpretation of extracted outcome: Refutational Synthesis (RS): Assessment of time to achieving implementation outcomes—overall progress or within individual modes.	Implementation pace	The pace of a given implementation effort (e.g., progression through implementation stages); measurement of speed evaluated in the implementation process based on - the time elapsed to achieve predefined implementation milestones, - the time elapsed to attain predefined implementation outcomes, - implementation progress between predefined time periods, - the rate of progress (or changes in slope) over time or between milestones, and - the pace of iterative development or improvement (Proctor 2022) (74)

The speed of implementation is an ambiguous concept that may refer to (i) the pace of research translation processes, (ii) the pace of implementation efforts, (iii) the pace of achieving service outcomes, and (iv) the pace of achieving clinical outcomes (74). The publication included in this review focuses on the conceptual development of pace in the first two situations. Including implementation pace as part of evaluating a given implementation effort may be appropriate in scenarios with a clear case or need to accelerate implementation efforts (such as during public health crises, e.g., during the COVID-19 pandemic). Incorporating the assessment of implementation pace may also be relevant when rapid and agile implementation approaches are used, where timely implementation of innovations is a critical component (75).

#### 3.2.4 Implementation adaptation

We propose *implementation adaptation* as the fourth attribute of the modes of implementation outcomes. Based on the synthesized conceptualizations of the related extracted outcomes, we define it as the extent to which implementation efforts are adjusted in response to needs and changes at the organizational/setting level or population level or in response to evolving knowledge during the life cycle of the innovation.

The individual extracted implementation outcomes linked to this attribute all overlap in their conceptualizations (Table 9): “adaptation”*—*defined as adapting implementation efforts related to contextual preparation that may involve modification to the innovation and/or the implementation strategies that enable the use of the innovation (56, 60, 70); “re-invention”—defined as the degree to which an innovation is modified by a user (32); and “evolvability”—defined as evolvability of innovations’ implementation across all phases, which may include adaptation (and potential de-implementation) in response to evolving and changing knowledge (46).

**Table 9:** Adaptation**—**as an attribute of the modes of implementation outcomes.

Attributes of the modes—adaptation	Extracted implementation outcomes	Definitions of extracted implementation outcomes (first author's name and publication year)
Synthesized interpretation of extracted outcomes: Line of Arguments (LOA): Adaptation is the extent of adjustments to implementation efforts to respond to changes and needs at the organizational, setting, or population level or to evolving knowledge during the life cycle of the innovation.	Adaptation	Innovation is developed, procedures are developed and revised, and people adapt to innovation/procedures (Gallivan 2001) (60). Adaptation for the setting and population (Bopp 2013) (70) Adaptation to context over time (Greenhalgh 2017) (56)
	Re-invention	The degree to which an innovation is changed or modified by a user during adoption and implementation (Rogers 2003) (32)
	Evolvability	“Evolvability” across the life cycle of innovations, including adaptation and potential de-implementation in light of changing and evolving evidence, contexts, and population needs—changes that may be reflected in adaptation to the innovation and/or the implementation strategies (Shelton 2020) (46)

Implementation adaptation as a process is an important consideration often part of implementation efforts. It helps ensure implementation flexibility and contextualization to improve the chances of implementation success (56). However, as an implementation outcome, it sheds light on gauging a balance between adaptation and fidelity (70).

#### 3.2.5 De-implementation

How should de-implementation be conceptually conceived in relation to implementation? Based on the synthesized conceptualizations, we identify *de-implementation* as an attribute of the modes of implementation outcomes that indicate the extent of unlearning outmoded practices by actively removing or replacing them. The publications included in this synthesis consider de-implementation either a standalone process/outcome or in conjunction with the overall implementation process of putting another innovation in place. Thus, de-implementation is classified into two types: disenchantment (i.e., active rejection) of an existing practice/previously adopted innovation (32, 76–81) or substitution (i.e., replacement) of an existing practice/previously adopted innovation with a newer innovation that is perceived to be better (32, 77, 80–82).

In both of these circumstances of whether de-implementation is triggered by disenchantment or substitution, a common feature of the extracted outcomes—“de-implementation” (77, 79–82), “de-adoption*”* (76, 78), and “discontinuance (replacement or disenchantment)” (32), which we map under the attribute “de-implementation”—is their focus on deliberate efforts to actively remove or replace (to some extent or completely) a previously implemented innovation that is no longer desired or needed (Table 10). However, not all the conceptualizations provided for these outcomes are directly translatable.

**Table 10:** De-implementation—as an attribute of the modes of implementation outcomes.

Attributes of the modes—de-implementation	Extracted implementation outcomes	Definitions of extracted implementation outcomes (first author's name and publication year)
Synthesized interpretation of extracted outcomes: Line of Arguments (LOA): Outcomes mapped to de-implementation overlap to indicate the extent of unlearning outmoded practices but delineate different aspects that reveal de-implementation as deliberate efforts to actively remove or replace an innovation that is no longer desired or needed De-implementation might be an isolated occurrence or jointly considered with active implementation efforts of another innovation.	De-adoption	A formal decision to de-adopt (discontinue use of) an innovation at any stage of implementation (Massatti 2008) (76) Discontinuation of a clinical practice after it was previously adopted (Niven 2015) (78)
	De-implementation	Divesting from ineffective and harmful medical practices informed by evidence to abandon or de-implement (Prasad & Ioannidis, 2014) (79) The use of low-value care is reduced or stopped on a structural basis in a planned process that uses a set of activities (van Bodegom-Vos 2017) (82) Discontinuation of interventions that should no longer be provided (McKay 2018) (77) The process of identifying and removing harmful and low-value practices (at any point during implementation) based on tradition and without scientific support; de-implemented practice may either be replaced with another practice based on evidence, modified as new evidence emerges, or removed entirely (Upvall 2018) (80). De-implementation is an implicit part of the implementation and organizational change (coupling de-implementation and implementation), which is associated with the unlearning process to discontinue or deviate from ineffective practice; it represents four types of change: partial reduction, complete reversal, substitution with related replacement, and substitution with unrelated replacement of existing practice (Wang 2018) (81)
	Discontinuance (replacement)	A decision to reject an idea in order to adopt a better idea that supersedes it (Rogers 2003) (32)
	Discontinuance (disenchantment)	A decision to reject an idea as a result of dissatisfaction with its performance (Rogers 2003) (32)

As shown in Table 10, some of these outcomes overlap in their conceptual definitions. However, some conceptualizations stopped at framing de-implementation as a standalone/isolated occurrence, implying it to be the opposite of implementation efforts (76, 78, 79). Others, however, go beyond to argue that de-implementation and implementation are not always opposites or mirror images but instead propose to conceptually, practically, and logistically couple or jointly consider de-implementation and implementation since de-implementation is an implicit part of implementation and organizational change in the general sense (81, 82). The main reason is the inherent unlearning when implementing new practices (77, 81, 82). Thus, considering de-implementation in conjunction with implementation efforts at various time points and modes (i.e., engagement, active implementation, and integration modes) could positively contribute to the implementation effectiveness of new practices. For example, Wang et al. stated that “coupling may result in effort-neutral change and considerably raise the likelihood for change, as intended” (81). McKay et al. also made a similar point by stating that in the face of technological and scientific advancement, “stopping [outmoded] existing practices to make room for better solutions becomes a necessity” (77).

As such, this conceptual synthesis of de-implementation suggests that the extent of unlearning has direct implications for achieving outcomes across the engagement, active implementation, and integration modes and the associated implementation outcomes when replacing an existing innovation or implementing another adjacent innovation within a setting. Thus, we suggest de-implementation as part of the attributes of modes of implementation outcomes to be integral to implementation efforts, given the risk of creating burdensome complexity by adding innovations to a service or system without, at the same time, removing other outmoded practices.

## 4 Discussion

**Figure 2:**
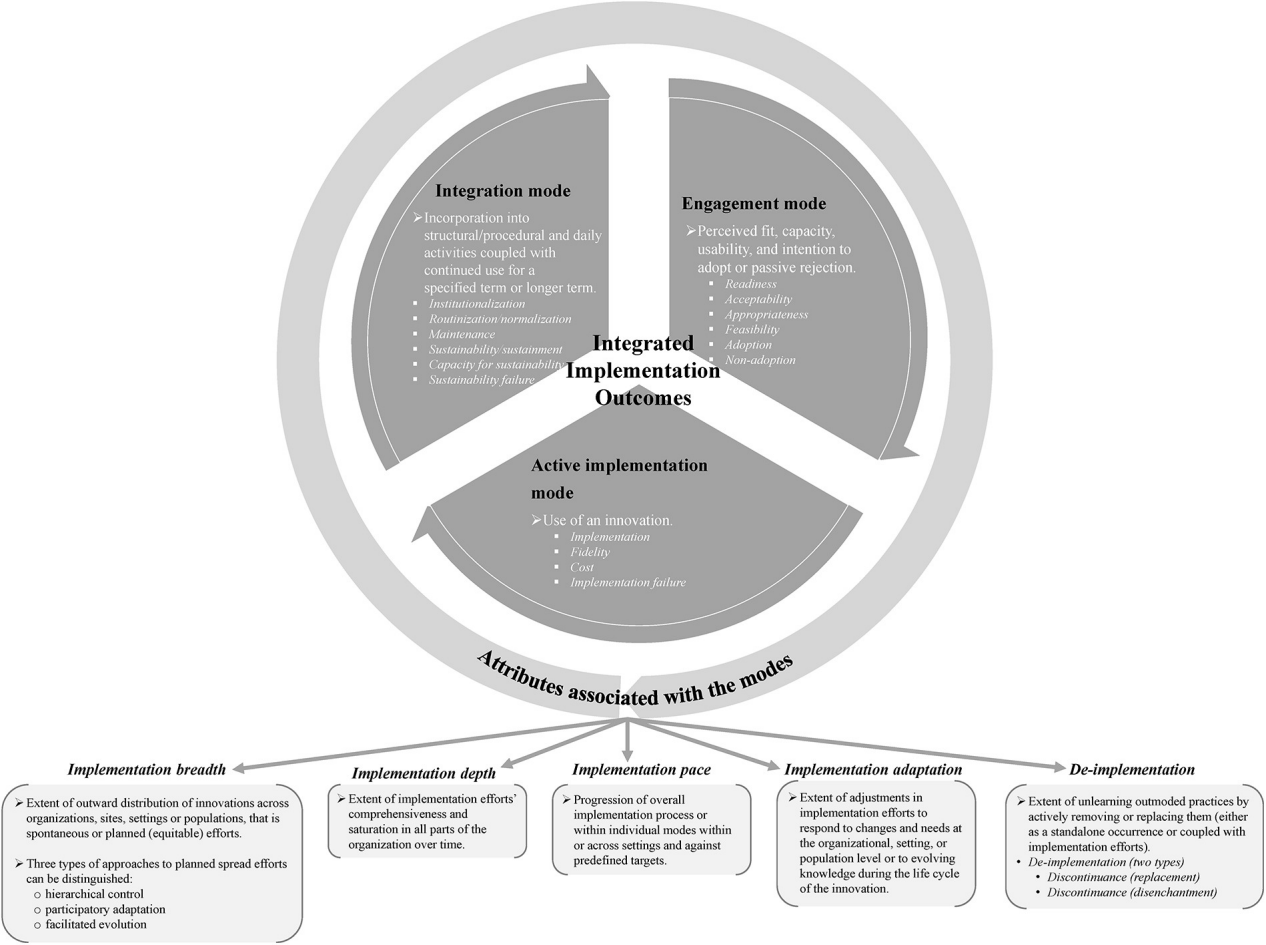
Integrated framework of implementation outcomes.

We have developed an integrated re-conceptualization of implementation outcomes of innovations (Figure 2). We conceptualize three *modes* of implementation outcomes (each refers to a distinct condition): engagement mode (perceived fit, capacity, usability, and intention to adopt or passive rejection of an innovation), active implementation mode (use of an innovation along with indications of the degree of fidelity, and cost of implementation), and integration mode (integration into structural/procedural and daily activities coupled with continued use over time). We further conceptualize five *attributes* associated with the modes of implementation outcomes: implementation depth (within a setting), implementation breadth (across settings), implementation pace (time to achieving implementation outcomes—overall progresses or within individual modes), implementation adaptation (responsive adjustments in implementation efforts at the organizational/setting/population level), and de-implementation (active removal or replacement efforts to unlearn outmoded practices that might be coupled with implementation efforts of another innovation). Together, the modes and attributes reveal an integrated framework of implementation outcomes that offers conceptual reductions of existing outcomes to clarify the relationships and distinctions between the individual outcomes in terms of translatability and complementary aspects.

As presented in Figure 2, the modes and attributes harmonize conceptual meanings of existing implementation outcomes by specifying the pertinence of the outcomes as either focal outcomes or outcomes characterizing the thoroughness of the implementation efforts. The engagement, active implementation, and integration modes delineate and structure relevant sets of implementation outcomes that we argue present core implementation outcomes of implementation efforts and are most salient under particular conditions during the implementation process journey, albeit being an iterative and not an entirely linear process. The focal implementation outcomes that are connected to the different modes are “readiness,” “acceptability,” “appropriateness,” “feasibility,” and “adoption” or “non-adoption” (*engagement mode*); “implementation,” “fidelity,” and “cost” or “implementation failure” (*active implementation mode*); and “institutionalization,” “routinization”/“normalization,” and “maintenance”/“sustainability”/“sustainment,” “capacity for sustainability,” or “sustainability failure” (*integration mode*). In comparison, the attributes of the three modes cut across the modes to specify the degree of implementation *depth*, *breadth*, *pace*, *adaptation*, and *de-implementation* achieved with implementation efforts.

### 4.1 Theoretical contributions

Our review contributes to theory in three ways. First, it clarifies and consolidates the diverse range of terminologies and conceptual meanings of implementation outcomes by considering their relatedness. We distinguished existing conceptualizations of implementation outcomes as either belonging to modes of implementation outcomes or serving as attributes of the modes. Within each mode or attribute, we translated similar outcomes into one another to achieve conceptual reductions of translatable terminologies and definitions or delineated the outcomes that partially overlap but reveal additional conceptual layers of the overarching mode or attribute. We further considered how the range of extracted outcomes contradict or refute one another to draw distinctions that elucidate the boundary conditions between the modes and attributes. It is important to distinguish outcomes for evaluating modes vs. attributes because the implementation outcomes linked to the modes present sets of outcomes fundamental to indicating the consequences of implementation efforts. The attributes reinforce the modes.

In addition, the modes are associated with distinct conditions of the implementation process that indicate the progression of the implementation efforts, while the attributes cut across the life cycle of the innovation. Such conceptual distinctions can help guide targeted ascertainment of the success or failure of implementation efforts. In contrast, conceptual explanations of outcomes specified in existing implementation outcome frameworks are dispersed across the diverse range of conceptual frameworks in our synthesis. For example, they commonly focus on addressing either short-term to mid-term implementation but not all aspects of the integration mode (e.g., short-term maintenance as well as longer-term sustainability) (46), implementation (15) but not de-implementation (22), or depth (local implementation) but not breadth (global spread) (21).

Second, we broadened the conceptualizations of implementation outcomes by conceptualizing the additions of overlooked or emerging outcomes absent in prominent implementation outcome frameworks. We distinguished the conceptualization of these additional conceptual outcomes by including them within the modes, e.g., assessment of readiness level, or as part of the attributes, i.e., pace, adaptation, and de-implementation. These additions consolidate and build on recent efforts to extend the RE-AIM and IOF frameworks (7, 15, 46, 74, 83). These insights address increasing recognition to redefine the scope of the outcomes to meet challenges related to evaluating implementation in complex healthcare systems and related key issues (8, 15, 19, 84). In the proposed integrated framework, we also included within the conceptualizations of the modes outcome concepts that may reveal abandonment of implementation efforts, i.e., non-adoption, implementation failure, or sustainability failure.

Third, we clarified and mapped the role of existing implementation outcomes in analyses or evaluations of implementation efforts by explicitly distinguishing how implementation outcomes relate to different modes and the corresponding individual outcomes within each mode. Thus, evaluating each mode can provide insights into the effectiveness of engagement, active implementation, or integration of an innovation. This insight extends prior work theorizing phases of the implementation process but does not explicitly specify which core implementation outcomes apply within each phase (referred to as “modes” in this review). Instead, prior work primarily focuses on identifying the temporal relationships between phases, often depicted linearly, in terms of associated processes and contextual factors (70, 85–87).

Although the inclusion of pace might suggest a linear progression of implementation efforts, its conceptual definition suggests otherwise: rather than being a strictly sequential progression, it refers to the overall speed of the implementation efforts or the rate of progression within individual modes. Furthermore, we classified pace as an attribute, meaning that it is not included among the outcomes proposed as core or focal implementation outcomes (i.e., not mapped into the modes) and is therefore not necessarily relevant in all circumstances. In contrast, pace might only be deemed of interest for inclusion in an evaluation when there is a clear purpose for doing so. In general, the role of temporality in relation to implementation strategies requires further development in implementation theories, models, and frameworks (88).

Overall, the standardization of the conceptual definitions of implementation outcomes sought in this synthesis review aims to clarify and integrate their generic conceptualizations. An underlying assumption of this approach is that the synthesized definitions and generic features of these outcomes should hold across contexts. A “concept” is generally universal and therefore applicable across contexts. Hence, heterogeneity of these outcomes may arise once they are operationalized in specific settings (as expected). Implementation practitioners can address such heterogeneity by ensuring the conceptual definitions remain consistent across contexts. In this way, they can strike a balance between internal validity—which accounts for local contextual elements, objectives, and priorities driving how outcomes are measured—and external validity—which preserves the conceptual meaning of each outcome.

Related to this point is the importance of high-quality instruments for measuring implementation outcomes, which is a necessary intermediary step (i.e., an interface) between the conceptual form of implementation outcomes and implementation strategies. Accordingly, when it comes to selecting concepts of implementation outcomes and their related instruments to assess which implementation strategies work best, we agree with recommendations from other researchers that the selection process be guided by the context and project objectives (16). In the proposed integrated framework, the term mode emphasizes the condition of the implementation process, not the stage or phase, which would imply a linear process. Bracketing implementation outcomes into distinct timeframes (such as phases or stages) is problematic since implementation processes typically unfold in parallel and iteratively. Thus, when considering which implementation outcomes might be relevant for a particular implementation project, a circumstance-driven approach (e.g., based on context and objectives) can better guide the selection of both the outcomes and a psychometrically robust instrument. However, the availability of instruments for implementation outcomes and their psychometric quality remains underdeveloped (16, 17, 89), with most existing tools concentrating on concepts such as acceptability, appropriateness, feasibility, adoption, fidelity, cost, penetration, and sustainability (16, 17, 89). Little or no development has been made for outcomes such as implementation depth, breadth, de-implementation, and implementation (the extent to which an innovation is in place).

Operationalization of the implementation outcomes into measures is crucial to enable practical and measurable observations and bridge the connection between conceptual implementation outcomes and implementation strategies. Once outcome measures have been developed, it becomes apparent how the conceptual implementation outcomes are directly related to the implementation strategies, which encompass implementation activities (16).

### 4.2 Practical implications

Our integrated implementation outcomes framework seeks to better reflect the complex reality of implementing innovations in health systems. It is thus in line with recent discussions that are moving away from conceptualizing the implementation process as a linear step-by-step or phased model to more iterative, integrated, or system-based models (8, 90). Our integrative re-conceptualization represents a more holistic and relational perspective on implementation outcomes, integrating focal outcomes used to assess the effectiveness or failure of implementation across three distinct modes of the implementation process on a scale of depth, breadth, and pace while also considering adaptation and de-implementation. Thus, by clarifying and identifying relevant outcomes for selection and operationalization in measurement, one of the implications of the framework we propose is its helpfulness in focusing the attention of evaluators to direct the scope of solutions or decisions that stem from evaluation outputs to improve or learn from implementation efforts. This curtails confusion and ambiguity of implementation outcomes, which are also observed to be used interchangeably in a way that blurs conceptual distinctions; it may also aid syntheses across research publications (14). However, we acknowledge that the interchangeability of some concepts, such as “sustainability” and “sustainment,” will likely persist. Sustainability is a dynamic concept (7, 47, 57, 58) that makes nuanced conceptual distinctions between the ability to predict and provide assurance of longer-term maintenance and sustainment of an implemented innovation explaining the actual continued delivery. In our review, to minimize blurred conceptual distinctions and encourage consistency in the language used, we denote the terms “sustainability” and “sustainment” (both used by authors) as representing the actual continued delivery of an innovation, which closely overlap with or might even be completely translatable with the conceptualization of “maintenance” (12, 46). In comparison, we denote and recommend using the term “capacity for sustainability” to represent the potential or likelihood of continued delivery.

Another implication of the clarified and simplified approach to conceptualizing implementation outcomes can also answer the call from practice and policy stakeholders for more pragmatic outcome measures (5). The number of outcomes is reduced and mapped accordingly under the three modes of implementation outcomes and the five associated attributes. Depicting the conceptualization of the outcomes this way makes their condition and saliency more straightforward, which addresses the recommendation to report the mode (so-called phase) during which relevant outcomes are observed or are most salient during the implementation process (14).

The proposed integrated framework of implementation outcomes in our review can be deployed as a complementary framework with other existing frameworks, e.g., determinant frameworks such as Consolidated Framework for Implementation Research (CFIR) (7, 91), implementation theories such as Normalization Process Theory (92), established implementation outcome measures (16, 17, 89, 93), and measures of implementation progress such as the Stages of Implementation Completion (68). It can particularly inform theory-based evaluations aimed at understanding and assessing the effectiveness of implementation efforts as a proximal indication to achieving implementation success, for example, evaluation designs using program theories or logic models such as realist evaluations (94–98).

### 4.3 Limitations and future research

Our goal was not to achieve comprehensiveness but to capture key implementation outcomes. However, there is a possibility that we missed publications on implementation outcome frameworks and publications that conceptually further develop individual outcomes. Nonetheless, we applied a rigorous interpretive synthesis approach that combined systematic search with purposive search, including hand-searching references, citation tracking, and targeted citation searching, as a best-fit approach to balance comprehensiveness and relevance to capture and include publications conceptualizing key outcomes.

To clearly define and differentiate “health service delivery of innovations,” we applied and defined the exclusion criteria; we excluded publications focusing on the implementation of innovations in a private for-profit health context, non-health service practice context, or only involving non-health service providers. Excluding these contexts limits the scope and transferability of our findings. However, we attempted to define clear eligibility criteria to focus the review on comparable data from the literature. To ensure that we included comparable publications, we excluded publications focusing on contexts and innovation types that would influence implementation outcomes and their conceptualizations in a very diverse way, potentially blurring the boundary condition of implementation outcomes included in our consolidated conceptualization. We expected implementation outcomes and their conceptualization to be very different between private/for-profit and public/not-for-profit contexts and very different between non-health service innovations (e.g., financial/policy/governance contexts) and health service delivery innovations. Future research could expand our eligibility criteria to explore other sectors, settings, and innovation types.

Future research could further develop the integrated re-conceptualization of implementation outcomes by refining, expanding, or testing the proposed framework. Refining or expanding the modes, attributes, or individual outcomes might be helpful to advance the conceptual meanings and their application in practice, particularly for outcomes with limited conceptual developments, such as implementation pace and equitable implementation. The assessment of equitable implementation, which we proposed as being part of the breadth of implementation efforts, is significant because it is a proximal indicator to track the achievement of health equity outcomes, which is an important concern in health systems. However, there has been insufficient conceptualization and operationalization of what it means to include an equity lens in implementation outcomes and service system outcomes, although it is an area that is receiving increasing attention (15, 46, 99–101). Thus, there are opportunities to identify additional emerging concepts that are more pertinent to key issues related to the implementation of health innovations.

Future research could also test novel aspects of implementation outcomes advanced in this review, such as the attributes, including depth and breadth of implementation efforts and de-implementation (when relevant), to understand their usefulness, applicability, and saliency across the modes of implementation outcomes.

### 4.4 Conclusion

The suggested re-conceptualization of implementation outcomes integrates concepts developed in extensive literature from various traditions and for different implementation efforts of health innovations and evaluation purposes, reflecting the complex reality of implementation practice. It provides much-needed re-conceptualization of existing outcomes, thereby clarifying and distinguishing between focal implementation outcomes organized as modes and outcomes that can be considered as attributes of the modes that signal the thoroughness of implementation efforts. It offers a holistic yet concise and more explicit structure and guidance for improving the development and application of measures to assess the implementation effectiveness of health innovations.
